# Triad of bladder agenesis with solitary kidney and ectopic ureter

**DOI:** 10.4103/0970-1591.44271

**Published:** 2008

**Authors:** Sajni I. Khemchandani

**Affiliations:** Consultant Pediatric Urologist, Part time Professor in Transplantation Surgery, Institute of Kidney Diseases and Research Centre, Dr. H L Trivedi Institute of Transplantation Sciences, Civil Hospital Campus, Ahmedabad, Gujarat, India

**Keywords:** Bladder agenesis, continent urinary diversion, ectopic ureter, solitary kidney

## Abstract

The bladder agenesis is an extremely rare congenital genitourinary anomaly; only 60 cases have been reported in the English literature and only 19 of these were noted in viable neonates.[1] Our case represents the 20th live birth with bladder agenesis. The triad of bladder agenesis with solitary kidney and ectopic ureter is seldom compatible with life due to associated anomalies.[2,3] Successful treatment and the long-term prognosis are usually poor because of the associated abnormalities. In our case, function of left solitary kidney was good and child did not have associated life-threatening disorder. Hence child was successfully managed with continent urinary diversion with good quality of life.

## INTRODUCTION

The triad of bladder agenesis with solitary kidney and ectopic ureter is an extremely rare congenital genitourinary anomaly. The vast majority of the viable children with this congenital anomaly are females and almost all of the individuals afflicted will have multiple associated orthopedic and neurologic anomalies and is seldom compatible with life. The purpose of this article is to elucidate the use of continent urinary diversion in this rare patient population with good quality of life.

## CASE REPORT

**Figure 1:**
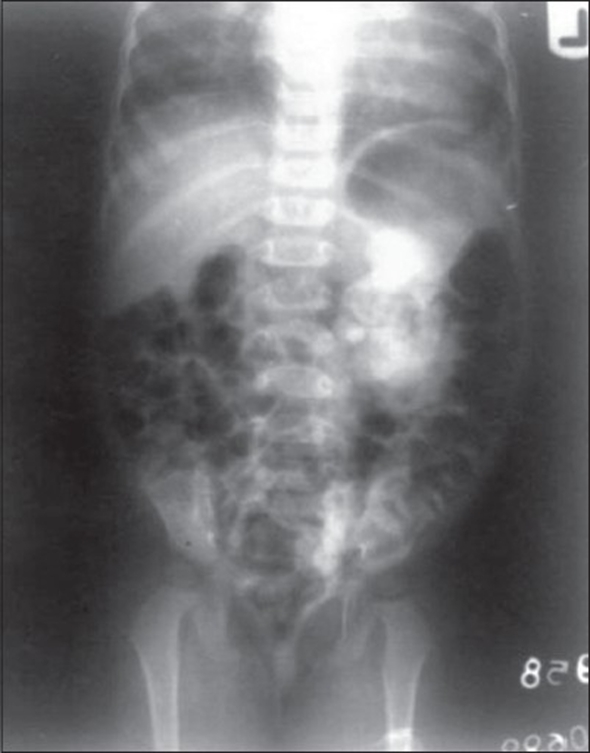
Intravenous pyelography (IVP) show left hydroureteronephrosis with nonvisualization of right kidney and urinary bladder

A 2-year-old female child presented with persistent dribbling incontinence and recurrent urinary tract infections. She has never passed urine normally in stream. Mother did not give any history of fever or drug intake during pregnancy. Antenatal ultrasonography did not diagnose any genitourinary anomaly. On examination, child was healthy, abdominal examination was also normal. External genitalia were normal except small pit like external urethral meatus with excoriations over vulva. There was no clinical indication of any other congenital anomaly. Her serum creatinine was 0.67 mg%. Urine examination showed 15-20 pus cells per high power field. Urine culture and sensitivity showed growth of *E. coli* organisms. Ultrasonography and intravenous pyelography showed mild hydroureteronephrosis of left kidney with nonvisualization of right kidney and urinary bladder [Figure 1]. Technetium 99m-diethylenetetraminepenta – acetic acid (DTPA) renal scan confirmed absence of right kidney and urinary bladder. Retrograde uretero-nephrogram showed left ectopic ureteric orifice opening in the vestibule with pit like external urethral meatus and absence of urinary bladder. The diagnosis of triad of bladder agenesis with solitary kidney and ectopic ureter was made.

**Figure 2:**
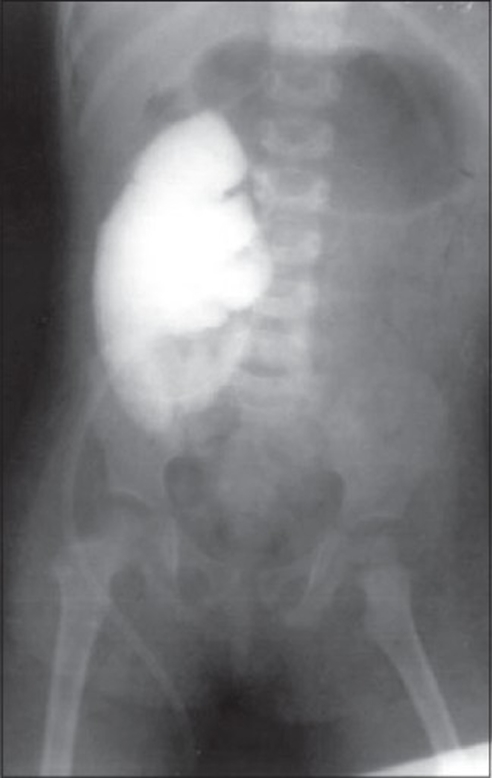
Pouchogram shows good capacity neobladder, with no evidence of reflux or urinary leak

The child was explored; continent ileocecal pouch, the Penn pouch was reconstructed with Mitrofanoff principle. The ileocecal pouch had ureter attached to it with a submucosal antireflux anastomosis. The appendix was then placed into another tenia with a flap-valve mechanism, and the cecal end of the appendix was brought to the umbilicus as a catheterizable stoma. Postoperatively, child was asymptomatic, pouchogram done at 3 weeks showed good capacity neobladder, with no evidence of reflux or urinary leak [Figure 2]. Child was kept on clean intermittent catheterization (CIC) through umbilical stoma. The child had total continence and ease of catheterization at 4-h interval between catheterizations, stable upper urinary tracts, and no incidence of pyelonephritis. Postoperative complications consisted of asymptomatic bacteriuria, and persistent mild metabolic acidosis. Child is on CIC and pouch washes with soda bicarbonate twice a week, with normal renal functions, with 2-3 episodes of asymptomatic bacteriuria over 4 years of follow-up.

## DISCUSSION

Bladder agenesis is an extremely rare congenital genitourinary anomaly; only 60 cases have been reported in the English literature and only 19 of these were noted in viable neonates.[1] The vast majority (90%) of the viable children with this congenital anomaly are females.[1] Almost all of the individuals afflicted will have multiple associated orthopedic and neurologic abnormalities.[2,3] The development of the bladder typically occurs at approximately 5 weeks’ gestation, with the division of the cloaca into the urogenital sinus and rectum. During cloacal division the distal portions of the mesonephric ducts are absorbed into the wall of the bladder. Bladder agenesis may be the result of secondary atrophy of the urogenital sinus, perhaps owing to the lack of distension with urine caused by failure of incorporation of the mesonephric ducts and ureters to develop into the trigone.[4,5] It may also represent the most severe form of ureteral ectopia.[6] In female patients with bladder agenesis, the ureters empty into the mullerian structures so they may terminate in the uterus, anterior vaginal wall, or vestibule. This allows preservation of renal function. While in the male patients with bladder agenesis, rectum,[2] or patent urachus is the only means of achieving urinary drainage. Other associated urinary anomalies are unilateral or bilateral renal agenesis, renal dysplasia, absence of seminal vesicles, and prostate. Our patient was also a female with unilateral renal agenesis with ectopic ureter opening into the vestibule with preservation of function in solitary kidney.

The management of an ectopic ureter and bladder agenesis depends, in part, on the associated anomalies. Most associated kidneys are ultimately removed because of dysplasia, hypertension, or infection. Successful treatment and the long-term prognosis are usually poor because of the associated abnormalities.[1]

Treatment options in this disorder include continent and noncontinent urinary diversion, either internally or externally.[6] The complete assessment of the child's physical status and the social situation dictates the management plans. In this case, the parents insisted on a continent urinary diversion and no contraindications to this procedure were present in the patient, except that child was very small. The continent ileocecal pouch, the Penn pouch with Mitrofanoff principle was reconstructed. In Penn pouch, the appendix serves as the continence mechanism and cecal end is used as catheterizable stoma. This procedure was popularized by Duckett and Snyder, Penn pouch with Mitrofanoff principle is simple and reliable technique.[7–9]

We have reviewed literature extensively; to date reconstruction of the lower urinary tract with the attainment of continence has not been described in cases of bladder agenesis.[5] Our case is unique; since function of left solitary kidney was good and child did not have associated life-threatening disorder. Hence, this child was managed successfully with continent urinary diversion. Although child is on CIC, her quality of life is good.
